# Effectiveness of Intravenous Cyclophosphamide in a Patient With Anti-amphiphysin Autoimmunity Presenting With Bulbar Palsy and Cerebellar Ataxia: A Case Report

**DOI:** 10.7759/cureus.65350

**Published:** 2024-07-25

**Authors:** Juri Nomoto, Hiroki Takatsu, Kazushi Yoshida, Haruka Matsuzawa, Shusaku Omoto

**Affiliations:** 1 Department of Neurology, The Jikei University Hospital, Tokyo, JPN; 2 Department of Neurology, The Jikei University Katsushika Medical Center, Tokyo, JPN; 3 Department of Respiratory Medicine, The Jikei University Katsushika Medical Center, Tokyo, JPN; 4 Department of Pathology, The Jikei University Katsushika Medical Center, Tokyo, JPN

**Keywords:** cyclophosphamide, cerebellar ataxia, bulbar palsy, non-stiff person syndrome, anti-amphiphysin antibody

## Abstract

Anti-amphiphysin antibody is a rare paraneoplastic autoantibody. A case of a 74-year-old man with anti-amphiphysin antibody and multiple symptoms, including bulbar palsy along with cerebellar ataxia, who responded to treatment with intravenous cyclophosphamide is reported. The patient presented with progressive unsteady gait and difficulty in swallowing food and water for three months. On admission, he had severe ataxia, downbeat and horizontal nystagmus, dysarthria, dysphagia, loss of tendon reflexes, and dysuria. Anti-amphiphysin antibodies were detected in the serum, resulting in the diagnosis of non-stiff anti-amphiphysin syndrome. No significant abnormalities were observed in imaging studies of the brain and the whole body. The patient was treated with high-dose intravenous immunoglobulin and steroids, yielding only slight improvement. After two courses of intravenous cyclophosphamide pulse therapy, his neurological symptoms, notably dysphagia and cerebellar ataxia, improved. Follow-up computed tomography and fluorodeoxyglucose-positron emission tomography/computed tomography showed enlarged mediastinal lymph nodes and hypermetabolic uptake of F-18 fluorodeoxyglucose six months after the onset of the neurological symptoms. Histological examination of a lymph node showed metastatic small cell lung cancer. This case highlights the efficacy of cyclophosphamide as second-line immunotherapy for anti-amphiphysin syndrome.

## Introduction

Anti-amphiphysin antibody is a rare paraneoplastic autoantibody detected in 0.06% of neurological patients whose sera were submitted for paraneoplastic autoantibody evaluation [1]. Anti-amphiphysin antibody was initially described in patients with paraneoplastic stiff-person syndrome [2]. Generally, neurological symptoms associated with anti-amphiphysin antibodies are highly diverse, and, therefore, it is difficult to establish a definitive early diagnosis. A retrospective case series of 63 patients with anti-amphiphysin autoimmunity showed that neurological presentations included neuropathy (52.4%), encephalopathy (30.2%), myelopathy (27%), stiff-person syndrome (28.6%), and cerebellar ataxia (17.5%) [1]. Cancer is detected in about 80% of patients with anti-amphiphysin antibodies, most commonly lung cancer (70%, mainly small cell lung cancer) and breast cancer (25%) [3]. However, when limited to cases of non-stiff anti-amphiphysin syndrome, cancer is detected in only 35% of cases [4]. A case of anti-amphiphysin antibody autoimmunity presenting with multiple symptoms, including dysphagia along with cerebellar ataxia, that responded to immunotherapy, especially cyclophosphamide, is reported. After treatment with immunotherapy, hidden small cell lung cancer was detected on follow-up computed tomography (CT) and fluorodeoxyglucose-positron emission tomography/computed tomography (FDG-PET/CT).

## Case presentation

A 74-year-old man with a history of type 2 diabetes mellitus and dyslipidemia presented with dizziness, unsteady gait, and difficulty in swallowing food and water for three months, which had been worsening over the preceding month. On admission, he had severe ataxia, downbeat and horizontal nystagmus in all directions of gaze, dysarthria, dysphagia, loss of tendon reflexes, and dysuria. His consciousness was clear, cognitive function was normal, and there was no muscle stiffness. Videoendoscopic examination of swallowing showed a pooling of saliva in the pyriform fossae and a diminished cough reflex. In the swallowing test, thickened water was not cleared from the hypopharynx by swallowing several times, and part of it was aspirated. On blood examinations, there were no abnormalities, including thyroid function and vitamin B1, vitamin B12, and folic acid levels. The antinuclear antibody titer was 1:80, and anti-GAD, anti-GM1, anti-GQ1b, anti-SS-A, anti-SS-B, and anti-AChR antibodies were negative. Cerebrospinal fluid (CSF) examination showed mild pleocytosis (6 cells/µL) (100% mononuclear cells), elevated protein levels (65.8 mg/dL), and a normal IgG index. Analysis of the CSF showed no infectious or malignant etiologies. Magnetic resonance imaging (MRI) of the head was unremarkable, and a (123I) N-isopropyl-p-iodoamphetamine (IMP) brain perfusion single photon emission computed tomography (SPECT) did not show perfusion abnormalities. At this time, subacute autoimmune-mediated cerebellitis with brainstem involvement was suspected. He was treated with intravenous immunoglobulin (IVIG) at a dose of 400 mg/kg for five days, but there was no improvement in the neurological symptoms. Two days after IVIG therapy, two courses of high-dose intravenous methylprednisolone, followed by oral prednisolone (1 mg/kg/day), were administered. Immunoblotting of serum by the EUROLineScan (EUROIMMUN, Lübeck, Germany) was positive (+++) for anti-amphiphysin antibodies and positive (+) for anti-SRY-box transcription factor 1 (SOX1) antibody (EUROLineScan flatbed scanner signal intensity: 0, 0-5; (+), 6-10; +, 11-25; ++, 26-50; +++, >50), whereas other antibodies associated with paraneoplastic neurological syndromes, such as anti-collapsin response-mediator protein-5 (CRMP 5)/CV2, anti-paraneoplastic antigen Ma2, ANNA type 2/Ri, anti-Purkinje cell cytoplasmic antibody type 1/Yo, anti-recoverin, anti-titin, anti-zic4, anti-glutamic acid decarboxylase 65 (GAD65), and anti-delta/notch-like epidermal growth factor-related receptor/Tr antibodies, were negative. The clinical and laboratory findings led to a diagnosis of non-stiff anti-amphiphysin syndrome with coexistence of anti-SOX1 antibody. Anti-amphiphysin antibody is a high-risk antibody for cancer [3]. To check for occult malignancies, serum tumor marker tests, whole-body CT, upper and lower gastrointestinal endoscopies, and gallium scintigraphy were performed, but no cancer was detected.

One month later, steroid treatment slightly improved both urinary retention and dysphagia, but the recovery in swallowing function was limited to oral ingestion of easy-to-swallow foods such as jelly, pudding, and mousse, and it did not result in the improvement of difficulty standing up and speech ability necessary for daily communications. Therefore, intravenous cyclophosphamide (500 mg/m^2^ of body surface/month) was started. After two courses of cyclophosphamide therapy, he was able to tolerate oral intakes such as easily mashed foods and soft foods. Moreover, he was able to stand up using a handrail and walk with a wheeled walking frame. In addition, his dysarthria and upper limb ataxia improved, enabling him to achieve intelligible speech and use his fingers to operate a smartphone precisely. He was discharged from the hospital three months after admission, and latent cancer was not detected on follow-up whole-body CT.

**Figure 1:**
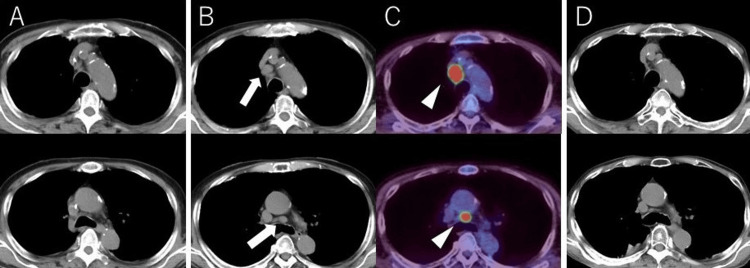
Axial images of serial unenhanced chest CT scans and axial PET-CT fusion. Chest CT scans on admission (A) showed no abnormalities. Follow-up chest CT scans soon after discharge showed enlarged mediastinal lymph nodes (B, arrows). One month after discharge, fluorodeoxyglucose PET-CT fusion showed increased uptake in the same region (C, arrowheads). After the chemoradiotherapy, the enlarged lymph nodes significantly reduced in size (D). CT, computed tomography; PET, positron emission tomography.

**Figure 2:**
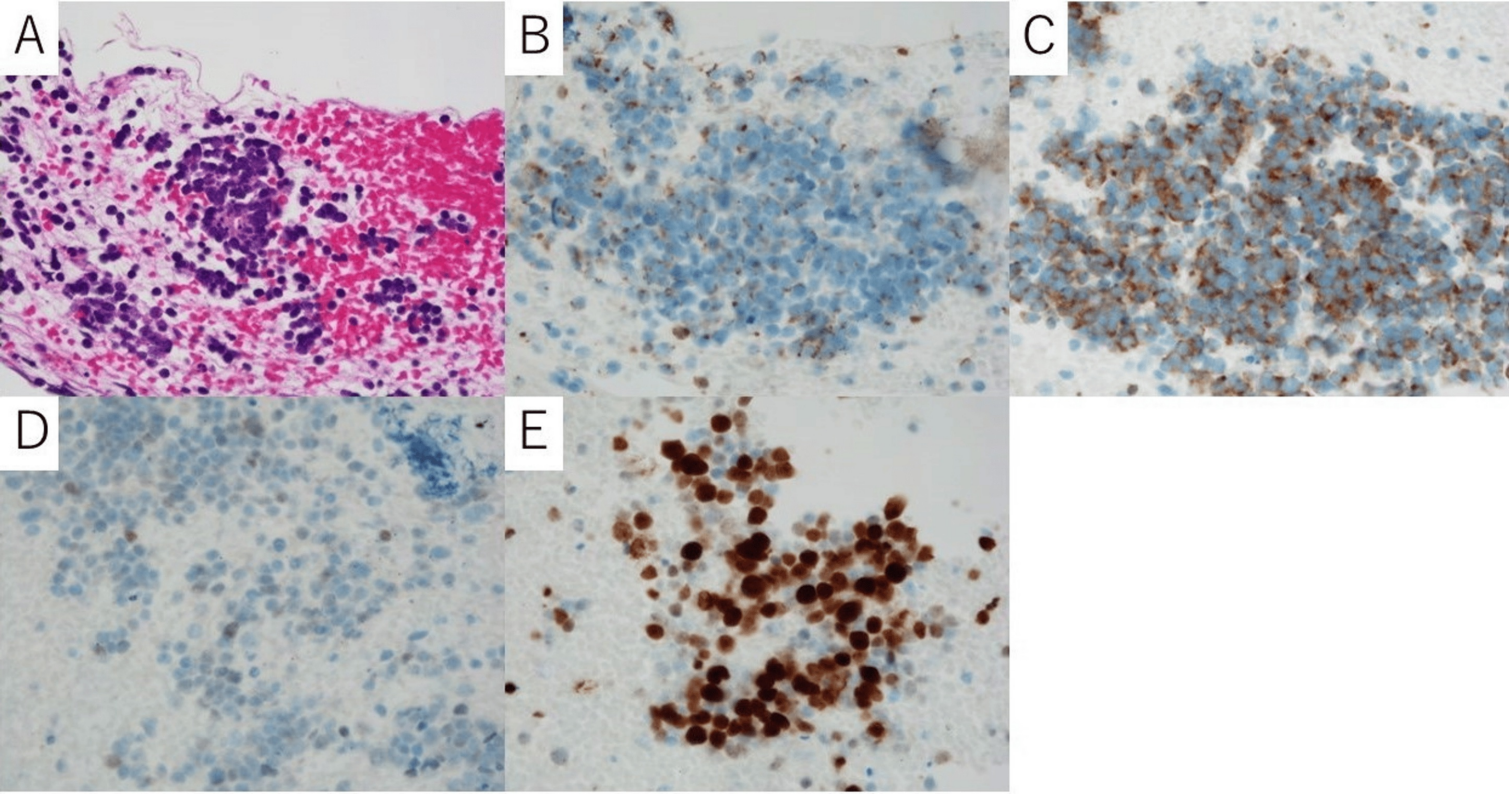
Pathological images of the lymph nodes. (A) Hematoxylin and eosin stain showed small round cells with scant cytoplasm arranged in loose clusters and focal rosette formation (original magnification ×600). (B) Cytokeratin AE1/AE3 stain was positive in a rim-and-dot-type pattern (original magnification ×600). (C) Synaptophysin stain was positive (original magnification ×600). (D) Thyroid transcription factor-1 stain was positive (original magnification ×600). (E) Ki-67 stain showed a high level of staining. The Ki-67 labeling index was 90% (original magnification ×600).

Whole-body CT on admission showed no obvious abnormalities (Figure 1A), whereas follow-up chest and abdominal CT showed enlarged mediastinal lymph nodes (Figure 1B) after discharge (six months after the onset of neurological symptoms). During the following sessions, FDG-PET/CT was performed, and further enlargement and fluorodeoxyglucose (FDG) uptake (maximum standard uptake value = 12.2) of the mediastinal lymph nodes were detected (Figure 1C). The lymph nodes were biopsied, and pathological examination showed small round cells with scant cytoplasm arranged in loose clusters and focal rosette formation (Figure 2A). Immunohistochemically, tumor cells were positive for cytokeratin AE1/AE3 (rim-and-dot-type pattern), synaptophysin, and thyroid transcription factor-1, and the Ki-67 (MIB-1) labeling index was 90% (Figures 2B-2E). Based on the biopsy examination results, small cell carcinoma was diagnosed. There was no evidence of distant metastatic disease. Therefore, the clinical staging for this patient was cTXN2M0 (stage IIIA). He received chemoradiotherapy with cisplatin (60 mg/m^2^, day one) and etoposide (80 mg/m^2^, days one to three) and accelerated hyperfractionated radiotherapy 45Gy/30fr. After the treatment, the target lesion was reduced (Figure 1D). On the other hand, his neurological symptoms showed no evident change. He is going to continue to receive several cycles of chemotherapy treatment according to the treatment protocol.

## Discussion

The present case showed the efficacy of cyclophosphamide therapy for cerebellar ataxia and bulbar palsy, and the latter is a rare manifestation in non-stiff anti-amphiphysin syndrome. Amphiphysin is a synaptic vesicle protein in presynaptic nerve terminals, which exists in GABAergic neurons with a widespread distribution. Anti-amphiphysin antibodies target intracellular antigens, associated with cytotoxic T-cell mechanisms [5], and anti-amphiphysin immunoglobulin G also causes presynaptic GABAergic inhibition. The widespread expression of GABAergic neurons and the presence of multiple immunological mechanisms are thought to be the pathogenesis of various neurological symptoms [6]. In the present case, immunoblotting of serum detected anti-amphiphysin antibodies very strongly, and the anti-SOX1 antibody was weakly positive. In the previous study, 74% of patients with anti-amphiphysin antibodies had one or more co-existing paraneoplastic antibodies [1]. Given the signal intensity on the serum immunoblotting test, the present patient exhibited the clinical presentation of anti-amphiphysin syndrome, although the co-existence of anti-SOX1 antibody may have had an effect on the neurological symptoms. Therefore, this is a valuable case with anti-amphiphysin antibodies, presenting multiple neurological symptoms, including bulbar palsy, cerebellar ataxia, autonomic neuropathy, and peripheral neuropathy.

According to previous reports, there have been only a few cases of non-stiff anti-amphiphysin syndrome patients presenting with dysphagia [4,7]. In reports of small case series, it was demonstrated that non-stiff person syndrome with anti-amphiphysin antibody showed limbic encephalitis (50%), dysautonomia (45%), cerebellar dysfunction (30%), brainstem encephalitis (20%), and peripheral neuropathy (20%) [4]. Dysphagia was reported in patients with brainstem encephalitis [4,7]. Wang et al. [7] reported that dysphagia can be attributed to targeting of the patient’s swallowing reflex in the central pattern generator and the motor component of the swallowing reflex located in the brainstem. Neuropathological studies of anti-amphiphysin autoimmunity showed a predominance of cytotoxic T-cells throughout the brainstem and spinal cord parenchyma [1]. Diffuse infiltration by cytotoxic T-cells in the brainstem may be associated with dysphagia.

Paraneoplastic antibodies against intracellular antigens such as anti-amphiphysin antibodies are generally less responsive to immunotherapy [5]. However, steroids for stiff-person syndrome and especially cyclophosphamide for neuropathy have been reported to be effective in patients with anti-amphiphysin autoimmunity [3]. One possible explanation for the effectiveness of immunotherapy is that amphiphysin autoimmunity appears to act through both cellular and humoral immunity, as mentioned above [6]. In a small case series, corticosteroids and/or IVIG were partially effective in seven of nine patients with non-stiff anti-amphiphysin syndrome presenting with encephalitis, especially in those without tumors [8]. In addition, in a small case series of non-stiff anti-amphiphysin syndrome, the most commonly used immunotherapy agents for first-line immunotherapy were steroids and/or IVIG [4]. Although immunotherapy showed good responses in most patients, additional treatments including rituximab and tacrolimus were used in cases of no response to steroids and IVIG. In one patient, who presented with limbic encephalitis, treatment with cyclophosphamide showed partial effectiveness. In the present case, IVIG and steroid therapy showed slight efficacy, whereas cyclophosphamide improved his neurological symptoms, especially bulbar palsy and cerebellar ataxia. Cyclophosphamide may help by inducing apoptosis and leading to fast and effective immunosuppression [9]. Intensive immunotherapy may be effective in paraneoplastic cases, even if the patient is later confirmed to have cancer. Also, early detection and treatment of the underlying cancer are of great importance for preventing further damage to the nervous system.

## Conclusions

A case of anti-amphiphysin antibody autoimmunity manifesting as the rare symptom of bulbar palsy along with cerebellar ataxia, which improved with cyclophosphamide, was presented. The pathogenesis of anti-amphiphysin autoimmunity involves cellular and humoral immune mechanisms, and active immunotherapy appears to be a good choice of treatment. Since non-stiff amphiphysin syndrome presents a diverse range of neurological symptoms, sometimes severe, cyclophosphamide might prove beneficial as a second-line treatment approach.
